# Translation, cross-cultural adaptation and validation of the Cincinnati prehospital stroke scale in Brazil

**DOI:** 10.1590/0004-282X-anp-2020-0246

**Published:** 2021-04-23

**Authors:** Priscila Masquetto Vieira de ALMEIDA, Rodrigo BAZAN, Octávio Marques PONTES-NETO, César MINELLI, José Eduardo CORRENTE, Gabriel Pinheiro MODOLO, Gustavo José LUVIZUTTO, Alessandro Lia MONDELLI

**Affiliations:** 1 Universidade Estadual Paulista, Faculdade de Medicina de Botucatu, Campus Botucatu SP, Brazil. Universidade Estadual Paulista Universidade Estadual Paulista Faculdade de Medicina de Botucatu Campus Botucatu SP Brazil; 2 Universidade de São Paulo, Faculdade de Medicina de Ribeirão Preto, Ribeirão Preto SP, Brazil. Universidade de São Paulo Universidade de São Paulo Faculdade de Medicina de Ribeirão Preto Ribeirão Preto SP Brazil; 3 Hospital Carlos Fernando Malzoni, Departamento de Neurologia, Matão SP, Brazil. Hospital Carlos Fernando Malzoni Departamento de Neurologia Matão SP Brazil; 4 Universidade Federal do Triângulo Mineiro, Uberaba MG, Brazil. Universidade Federal do Triângulo Mineiro Universidade Federal do Triângulo Mineiro Uberaba MG Brazil

**Keywords:** Stroke, Early Diagnosis, Emergency Medical Service, Sensitivity and Specificity, Acidente Vascular Cerebral, Diagnóstico Precoce, Serviços Médicos de Emergência, Sensibilidade e Especificidade

## Abstract

**Background:**

Use of internationally standardized instruments to assist healthcare professionals in accurately recognizing stroke early is recommended. The process of translation and cross-cultural adaptation is important for ensuring that scales are interpreted in the same way in different languages, thus ensuring applicability in several countries.

**Objective:**

To translate into Brazilian Portuguese, cross-culturally adapt and validate the Cincinnati Prehospital Stroke Scale, using a representative sample of the Brazilian population.

**Method:**

The present study included patients with suspected stroke who were treated at a Brazilian emergency medical service and referred to a stroke center. A systematic process of translation and cross-cultural adaptation of the original scale and application of the final instrument was performed. Statistical analysis was used to assess the sensitivity, specificity and accuracy of the scale. Cohen’s kappa coefficient was used to assess inter-rater reliability.

**Results:**

After translation and cross-cultural adaptation, the scale was applied to 64 patients. It showed 93.0% accuracy and 92.4% sensitivity in relation to the final “gold standard” diagnosis. Cohen’s kappa coefficient was calculated using data from 26 patients (40.6%) and showed excellent inter-rater reliability between items on the scale (0.8385 to 1.0000).

**Conclusion:**

The scale demonstrated excellent accuracy, sensitivity and inter-rater reliability. It was a useful tool for assisting healthcare professionals during initial assessments on patients with suspected stroke and significantly contributed to early recognition of stroke in a simple and quick manner.

## RESUMO

**Introdução:**

O uso de instrumentos padronizados internacionalmente para auxiliar os profissionais de saúde a reconhecer precocemente o AVC é recomendado. O processo de tradução e adaptação transcultural tem sido importante para garantir que a escala seja interpretada da mesma maneira em diferentes idiomas, assegurando sua aplicabilidade em vários países.

**Objetivo:**

Traduzir para o idioma português do Brasil, adaptar transculturalmente e validar a *Cincinnati Prehospital Stroke Scale* na população brasileira.

**Método:**

O estudo incluiu pacientes com suspeita de acidente vascular cerebral (AVC) atendidos por um Serviço de Atendimento Móvel Urgência (SAMU) 192 e encaminhados para uma Unidade de Cuidado Integral ao AVC. Foram realizados um processo sistemático de tradução e adaptação transcultural da escala original e a aplicação do instrumento final, que possibilitou a realização dos testes de sensibilidade, especificidade e acurácia, de acordo com o estudo original, além do índice de Cohen de Kappa, para avaliar a confiabilidade interobservador.

**Resultados:**

Após a tradução e a adaptação transcultural, a escala foi aplicada em 64 pacientes, apresentando acurácia de 93,0% e sensibilidade de 92,4% em relação ao diagnóstico final, considerado padrão-ouro. Em 26 pacientes (40,6%) foi possível calcular o índice de Cohen de Kappa, evidenciando excelente confiabilidade interobservador entre os itens da escala (0,8385 a 1,0000).

**Conclusão:**

A escala apresenta ótima acurácia, sensibilidade e concordância interobservador. Trata-se de um instrumento útil para auxiliar os profissionais da saúde durante a avaliação inicial do paciente com suspeita de AVC, uma vez que contribui significativamente para o reconhecimento precoce da doença de uma maneira simples e rápida.

## INTRODUCTION

Among chronic, non-communicable diseases, stroke is one of the main causes of death and neurological disability worldwide. Stroke can occur through two mechanisms: occlusion, leading to ischemic stroke; or transient ischemic attack and rupture of vascular blood vessel(s), leading to hemorrhagic stroke^1^^,^^2^.

Stroke is a disease heavily influenced by socioeconomic factors, reflected in the fact that the highest incidence rates are in underdeveloped and developing countries. In Brazil, stroke is one of the main causes of mortality; however, paradoxically, it is one of the most neglected diseases. Data released by the Brazilian Ministry of Health demonstrated a mortality rate of 56.58/100,000 inhabitants in 2017, and stroke ranked second among the most prevalent causes of death in the country^1^^,^^3^^,^^4^.

Ischemic stroke is the most common type of stroke in the population. In such cases, one of the most effective treatments is thrombolytic therapy using intravenous recombinant tissue plasminogen activator, which should be administered within 4.5 h of the onset of symptoms. This treatment yields excellent results and has reduced the number of deaths and disability. However, the proportion of patients who arrive at a hospital in a timely manner and receive treatment remains low (approximately 1 to 8%)^5^^,^^6^^,^^7^. Another evolving treatment that is gaining supportive evidence for its effectiveness is mechanical thrombectomy, which can be performed 6 to 24 h after the onset of symptoms. It involves physical removal of the thrombus using an endovascular device to restore cerebral blood flow. It is a treatment that reduces mortality and disability among stroke patients. Regardless of the type of treatment administered, studies have associated success of treatment with patients’ early arrival at the emergency room, soon after the onset of symptoms^8^^,^^9^.

In this scenario, prehospital care has become an important factor in treating stroke. This has been associated with an increased rate of patients who arrive at a hospital early. One of its highlights is the readiness of teams who have been trained to recognize early symptoms of stroke and appropriately refer patients to hospitals with specialized personnel and facilities, thus optimizing treatment. In regions where there are well-structured primary healthcare services with priority in referring suspected cases to a specialized hospital, thrombolytic therapy rates can reach 24%^7^^,^^10^.

Thus, to be successful in this process, it is important to qualify prehospital teams, especially in relation to early identification of the disease. The American Heart Association and European Stroke Organisation recommend continuing education programs for these teams and use of internationally standardized instruments to assist healthcare professionals in accurately recognizing stroke early^11^^,^^12^.

Worldwide, there are several such standardized instruments^13^. In Brazil, the Cincinnati Prehospital Stroke Scale (CPSS) is in widespread use among emergency sectors. This scale assesses three changes: facial paresis, and motor and speech changes. It demonstrates sensitivity between 79 and 95%, and specificity between 24 and 56%. However, these figures come from studies conducted in other countries^13^^,^^14^^,^^15^. It is a simple instrument, very similar to the FAST scale (i.e., Face, Arm, Speech and Time), which is also used internationally and has been associated with increased treatment rates^11^^,^^16^.

To our knowledge, however, no studies have assessed the applicability of CPSS to the Brazilian population. Thus, there is a need for a systematic process of translation and cultural adaptation of this scale, along with statistical analysis on its application. Accordingly, the objective of the present study was to translate the CPSS into Brazilian Portuguese, and then to cross-culturally adapt and validate it using a representative sample of the Brazilian population.

## METHODS

In this prospective study, the CPSS instrument was translated, adapted and validated. The study was conducted in a city located in the interior of the state of São Paulo, Brazil, where there are integrated prehospital care services and a stroke center. Both of these are certified by the Ministry of Health as part of the national urgency and emergency policy. The prehospital service is named “SAMU 192” and teams are divided into basic life support teams (nursing technicians and drivers) and advanced life support teams (physicians, nurses and drivers)^17^.

This study was performed in two phases. The first consisted of a systematic process of translation and cross-cultural adaptation of the original scale and application of the final instrument. The second phase consisted of statistical analysis to assess sensitivity, specificity and accuracy, in accordance with a previous study. The inter-rater reliability of the translated scale was assessed using Cohen’s kappa coefficient.

The CPSS^14^ consists of three items elaborated from the National Institutes of Health Stroke Scale (NIHSS). It assesses the presence or absence of facial paralysis, weakness in the arms and abnormalities of speech^14^.

### Translation and cross-cultural adaptation

This part of the study involved 10 participants who are recognized in their professional fields, including neurologists and clinicians, nurses, physiotherapists and native and Brazilian translators. They performed the translation and cross-cultural adaptation process in accordance with the methodology described by Beaton et al. 2007^18^, in five phases, as follows:

- Initial translation: The initial translations were performed by two bilingual translators (T1 and T2), whose native language was Brazilian Portuguese (i.e. the target language). They had different levels of experience: T1 was a professional with knowledge in the field of healthcare; and T2 was a professional in another field. Neither T1 nor T2 had seen the original instrument.
- Synthesis of translations: The initial translations were analyzed and synthesized into a single version (T12), after careful analysis of divergences between the versions and resolution of problems.
- Back-translation: Two professionals translated the T12 version back into the original language. This phase was intended to ensure that the T12 version reflected the same content as the items in the original version. Through this, two versions were generated: BT1 and BT2. This phase aimed to ensure that T12 reflected the same meaning as in the original version.
- Expert committee analysis: This committee consisted of researchers and healthcare professionals, in addition to the translators involved in the previous stages, and it consolidated all versions of the scale (i.e. T1, T2, T12, BT1 and BT2). Equivalence in semantics, and idiomatic, conceptual and experiential factors, and the content for analysis of the instrument, were considered in the analysis.
- Pre-test of the final version: the researcher interviewed the contributing professionals regarding possible doubts about the meaning of each item.

**Figure 1.**
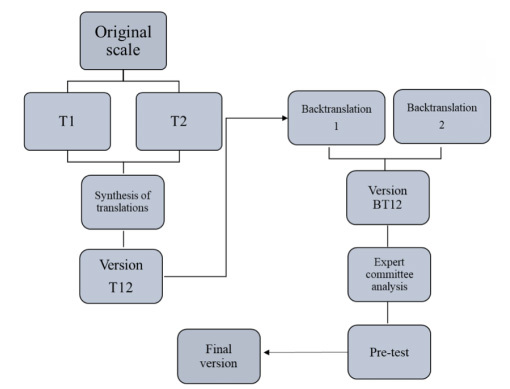
Methodological scheme used in the translation and cross-adaptation of the Cincinnati Prehospital Stroke Scale. Brazil, 2019.

The methodological scheme used in this process is shown in Figure 1.

### Application of the translated scale and cross-cultural adaptation

This stage was performed between October 2016 and December 2017. The final scale was applied by a SAMU 192 team. Nineteen professionals participated in this stage: eight doctors, six nurses and five nursing technicians. Application was implemented when the team arrived at the emergency site in cases of patients with suspected stroke to which the team had been alerted by the call center. Each professional was responsible for applying the scale to each patient. In cases in which there were two professionals in the same service, both performed patient evaluations and applied the scale independently, so that there was an opportunity to assess inter-rater reliability. All patients were referred to the stroke center.

At this stage, individuals >18 years of age who were suspected of experiencing stroke, and who were accompanied by an individual who agreed to authorize the use of patient data for research purposes, were included in the study. In addition to the data from application of the final scale, data from the prehospital and hospital records were also collected.

### Statistical analysis

To verify the reliability of the instrument, inter-rater reliability tests were performed using Cohen’s kappa coefficient among the professionals who performed prehospital care for the patient, considering the following values: <0.20, poor agreement; 0.20-0.39, fair agreement; 0.40-0.59 moderate agreement; 0.60-0.79, substantial agreement; and >0.8, almost perfect agreement^19^. For this analysis, scales that were applied concurrently by two professionals on the same patient were considered, which was possible in 26 cases (40.6%).

To assess the validity of the scale, the sensitivity, specificity, accuracy, positive predictive value (PPV) and negative predictive value (NPV) were calculated for all the instruments applied, considering a 95% confidence interval (95%CI). The final diagnosis, which was made at the hospital by a neurologist trained in stroke, with the aid of computed tomography, was taken to be the “gold standard” for all statistical tests. The term “stroke mimics” was used to classify patients who were not diagnosed with stroke^20^. The statistical analysis was performed using SAS version 9.4 (SAS Institute, Cary, NC, USA) for Windows (Microsoft Corporation, Redmond, WA, USA).

Ethics approval was granted by the Botucatu Medical School Research Ethics Committee.

## RESULTS

**Figure 2.**
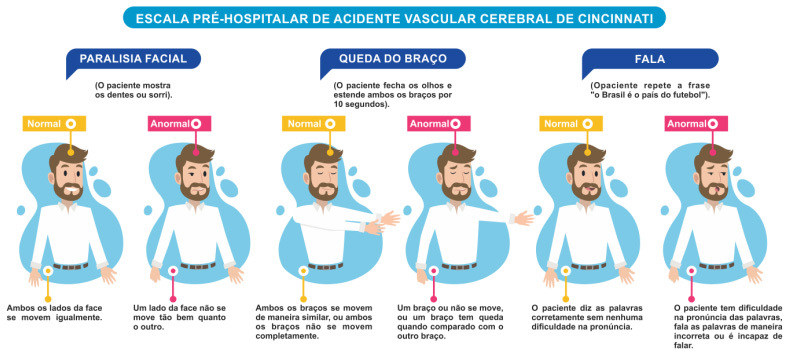
Translated version of the Cincinnati Prehospital Stroke Screen for use in Brazil. Brazil, 2019.

In the first step of this study, translation and cross-cultural adaptation of the original instrument were performed. In the process of translation into Brazilian Portuguese, difficulty was encountered with the words “droop” and “slur” because these have several meanings in the target language. However, the translation of these words was resolved after several discussions and, finally, a consensus was reached among the expert committee members. In the process of cross-cultural adaptation, the phrase “the sky is blue in Cincinnati” was changed to “Brazil is the country of football”, to facilitate patient understanding and, by extension, the feasibility of applying the instrument because the original expression is not culturally relevant in the Brazilian context. The original scale was thus translated and adapted for use in the Brazilian Portuguese language (Figure 2).

After this step, the final scale was applied to the target population, consisting of 64 patients with suspected stroke, of whom 45 (70.3%) had a confirmed diagnosis: 37 (82.2%) of these were classified as presenting ischemic stroke; 6 (13.4%), hemorrhagic stroke; and 2 (4.4%), an attack of transient ischemic disease. The data collected enabled statistical analysis on the use of the scale in a representative sample of the Brazilian population.

The results demonstrated high inter-rater reliability, as seen from the high value of Cohen’s kappa coefficient, especially for the items “arm drop” and “speech”, which reached the maximum value. Although there were small variations in agreement between the observers for the item “facial paralysis”, agreement remained high (0.8385), as shown in Table 1.

**Table 1.** Cohen's kappa coefficient for the final version of the Cincinnati Prehospital Stroke Scale, translated and adapted for use in the Brazilian Portuguese language. Brazil, 2019.

Scale item	k	95%CI
Facial droop	0.8385	0.6263-1.0000
Arm drift	1.0000	1.0000-1.0000
Speech	1.0000	1.0000-1.0000

95%CI: 95% confidence interval; k: Cohen's kappa coefficient.

The statistical tests revealed accuracy of 93.0% (95%CI 87.8-98.2), sensitivity of 92.4% (95%CI 86.0-98.8; PPV, 71.8) and specificity of 4.0% (95%CI 0-11.7; NPV, 16.7), in relation to the final gold standard diagnosis. The sensitivity of the scale increased according to the number of changed items, as follows: 1 abnormal variable, sensitivity=61.0% (p=0.5429); 2 abnormal variables, sensitivity=77.0% (p=0.0864); and 3 abnormal variables, sensitivity=88.0% (p=0.4660).

## DISCUSSION

In the present study, we translated and performed cross-cultural adaptation of the CPSS for use in the Brazilian Portuguese language, and then applied it to a sample target population and analyzed the results obtained. The scale demonstrated its utility in facilitating early diagnosis of stroke, yielding excellent values for sensitivity, accuracy and inter-rater reliability.

Worldwide, the process of cross-cultural adaptation has been important to ensure that scales are interpreted the same way in different languages, thus ensuring applicability in several countries^18^^,^^21^. However, there is currently no consensus in the literature regarding the strategies that should be used. Nevertheless, it is clear that it is a complex process that requires methodological rigor to guarantee the semantic, idiomatic and conceptual equivalence of the scale in question. In the present study, we opted to follow the theoretical framework described by Beaton et al.^18^, which provides recommendations for the process of translation and transcultural adaptation of scales. This proposes a process that encompasses five phases: initial translation, synthesis of translations, back-translation, analysis by an expert committee and pre-test of the final version. All the steps in the translation and cross-cultural adaptation of the CPSS were performed, and there was a need to change some terms for cross-cultural adaptation of the scale, to ensure quality and applicability to the Brazilian population.

After the process of translation and cross-cultural adaptation, the final scale was applied to patients with suspected stroke, who were treated by a SAMU 192 team and referred to a stroke center. This made it possible to analyze the applicability of the scale. The results from the analysis revealed that the majority of the patients examined were diagnosed with ischemic stroke, which corroborated global statistics that have classified this as the most prevalent type of stroke among the world’s population^22^. There is a need to improve the quality of early recognition of stroke and the referral of patients to a qualified hospital, given the extremely time-sensitive nature of stroke. The percentage of patients receiving treatment in Brazil remains low, mainly due to delays in arrival at a hospital. This makes it essential for the healthcare professionals involved in prehospital care to recognize the signs and symptoms of stroke, and to implement protocols for proper referral^10^.

Inter-rater reliability was analyzed using Cohen's kappa coefficient. This analysis was performed in 40.6% of the cases, for whom there was the possibility of applying the scale by two professionals concurrently. There was slight variation in concordance regarding the facial paralysis item, thus demonstrating difficulty in assessing this item, which had also been reported in another study^23^. Nevertheless, the values found were considered to be excellent, and did not hinder the use of this scale.

The latest systematic review to assess the use of instruments for early stroke recognition^11^ reported that application of the CPSS in several countries had demonstrated sensitivity values between 44 and 95%. Considering the data reported in the international literature, it was evident that the scale translated and adapted for use in the Brazilian population demonstrated high sensitivity (92.4%), although it demonstrated low specificity (4.0%), which was also consistent with the literature^11^^,^^12^^,^^13^. In addition, the accuracy of the scale was high (93.0%), which reflected its excellent accuracy in diagnosing the disease.

It was noted that the sensitivity increased according to the number of items that presented changes, such that the greater the number of changes was, the greater the risk was that the patient was actually having a stroke. In this regard, the scale is an important instrument for systematizing patient care, thereby increasing the chances of early diagnosis.

The results from the present study revealed that the scale demonstrated excellent accuracy, sensitivity and inter-rater reliability. The CPSS can assist professionals in the emergency sectors during the initial assessment of patients with suspected stroke, and can significantly contribute to early recognition in a simple and fast manner. Because of its simplicity and objectivity, application of this scale becomes useful for the entire healthcare team working in prehospital care, and not just the medical professionals. Considering the configuration of Brazilian prehospital care, which is formed mostly by professionals from nursing teams, this scale can have a positive impact on making early diagnoses of stroke and increasing the rates of appropriate and timely treatment.

There were limitations to the present study. The first of these was our inability to assess intra-observer reliability, because prehospital care does not allow application of the scale at two times by the same evaluator. However, we believed that this can be analyzed during hospital care, given that there is a longer period of contact with the patient. In addition, we were not able to analyze inter-rater reliability in all cases because only cases that were attended by the advanced support unit allowed application by two professionals simultaneously. Nevertheless, we believe that other studies may confirm this result, which was an excellent rate, despite only being assessed in 40.6% of the sample.

In conclusion, the CPSS was a useful scale for helping healthcare professionals in the emergency sectors during the initial evaluation on patients with suspected stroke. It significantly contributed to early recognition of stroke in a simple and quick manner, and showed excellent accuracy, sensitivity and inter-rater reliability.
